# The ecdysteroid receptor regulates salivary gland degeneration through apoptosis in *Rhipicephalus haemaphysaloides*

**DOI:** 10.1186/s13071-021-05052-2

**Published:** 2021-12-20

**Authors:** Xiaojuan Lu, Zhipeng Zhang, Dongqi Yuan, Yongzhi Zhou, Jie Cao, Houshuang Zhang, Itabajara da Silva Vaz, Jinlin Zhou

**Affiliations:** 1 Key Laboratory of Animal Parasitology of Ministry of Agriculture, Shanghai Veterinary Research Institute, Chinese Academy of Agricultural Sciences, Shanghai, 200241 China; 2 Centro de Biotecnologia, Universidade Federal Do Rio Grande Do Sul, Porto Alegre, RS Brazil

**Keywords:** Tick, *Rhipicephalus haemaphysaloides*, Ecdysone receptor, Ultraspiracle, Salivary gland degeneration, Apoptosis

## Abstract

**Background:**

It is well established that ecdysteroid hormones play an important role in arthropod development and reproduction, mediated by ecdysteroid receptors. Ticks are obligate hematophagous arthropods and vectors of pathogens. The salivary gland plays an essential role in tick growth and reproduction and in the transmission of pathogens to vertebrate hosts. During tick development, the salivary gland undergoes degeneration triggered by ecdysteroid hormones and activated by apoptosis. However, it is unknown how the ecdysteroid receptor and apoptosis regulate salivary gland degeneration. Here, we report the functional ecdysteroid receptor (a heterodimer of the ecdysone receptor [EcR] and ultraspiracle [USP]) isolated from the salivary gland of the tick *Rhipicephalus haemaphysaloides* and explore the molecular mechanism of ecdysteroid receptor regulation of salivary gland degeneration.

**Methods:**

The full length of *RhEcR* and *RhUSP* open reading frames (ORFs) was obtained from the transcriptome. The RhEcR and RhUSP proteins were expressed in a bacterial heterologous system, *Escherichia coli*. Polyclonal antibodies were produced against synthetic peptides and were able to recognize recombinant and native proteins. Quantitative real-time PCR and western blot were used to detect the distribution of RhEcR, RhUSP, and RhCaspases in the *R. haemaphysaloides* organs. A proteomics approach was used to analyze the expression profiles of the ecdysteroid receptors, RhCaspases, and other proteins. To analyze the function of the ecdysteroid receptor, RNA interference (RNAi) was used to silence the genes in adult female ticks. Finally, the interaction of RhEcR and RhUSP was identified by heterologous co-expression assays in HEK293T cells.

**Results:**

We identified the functional ecdysone receptor (RhEcR/RhUSP) of 20-hydroxyecdysone from the salivary gland of the tick *R. haemaphysaloides*. The *RhEcR* and *RhUSP* genes have three and two isoforms, respectively, and belong to a nuclear receptor family but with variable N-terminal A/B domains. The *RhEcR* gene silencing inhibited blood-feeding, blocked engorgement, and restrained salivary gland degeneration, showing the biological role of the *RhEcR* gene in ticks. In the ecdysteroid signaling pathway, *RhEcR* silencing inhibited salivary gland degeneration by suppressing caspase-dependent apoptosis. The heterologous expression in mammalian HEK293T cells showed that RhEcR1 interacts with RhUSP1 and induces caspase-dependent apoptosis.

**Conclusions:**

These data show that RhEcR has an essential role in tick physiology and represents a putative target for the control of ticks and tick-borne diseases.

**Graphical Abstract:**

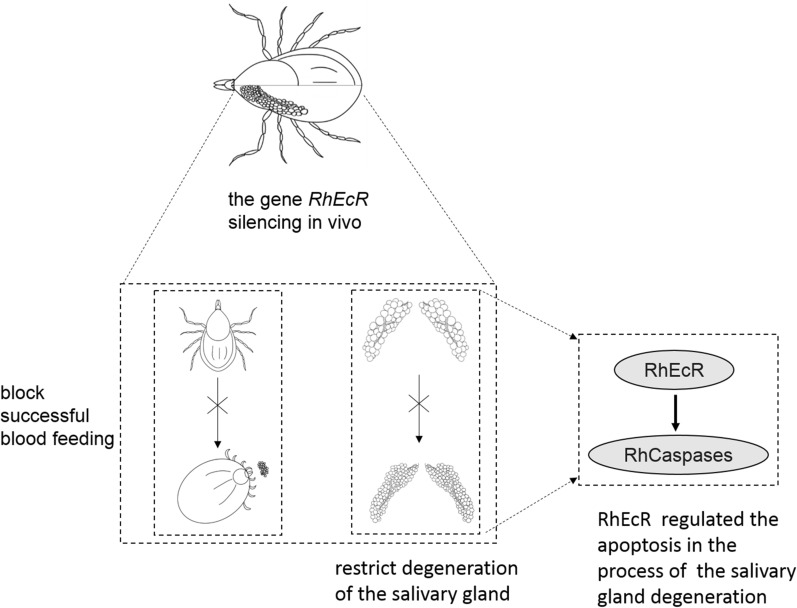

**Supplementary Information:**

The online version contains supplementary material available at 10.1186/s13071-021-05052-2.

## Background

Ticks are obligate hematophagous arthropods and vectors of many pathogens including fungi, protozoa, rickettsias, viruses, parasites, and bacteria [1–3]. Ticks are the most important vectors of pathogens that affect animals and are second only to mosquitoes in pathogen transmission to humans [4]. Ticks undergo continual differentiation and remodeling of tissues while molting from larvae to nymphs and from nymphs to adults [5], as well as during and after the blood-feeding period.

Because of the long periods of blood-feeding [6], the tick salivary gland undergoes extensive and orderly differentiation to maintain the tick–host feeding interface [7]. In female ticks, three morphologically distinct acini types are present in the salivary gland. Type I acini is associated with off-host osmoregulation. Types II and III are involved in the synthesis and secretion of protein factors and fluid secretion [8, 9]. It was reported that the ultrastructure of type I, II, and III acini in ixodid ticks revealed no changes in the numbers and the types of cells during the course of feeding [8]. However, during feeding, the salivary gland of adult ixodid ticks undergoes remarkable growth and differentiation, with acini types II and III showing the greatest morphological changes [9]. These changes are accompanied by an increase in the rate of protein synthesis [10, 11]. The volume of type II and III acini increases during the blood-feeding process but decreases upon engorgement and salivary gland degeneration [12]. Secreted tick saliva contains substantial amounts of hemostatic and immunomodulatory molecules that are essential for blood-feeding behavior [12]. The saliva is also involved in pathogen transmission to the vertebrate host. Many morphological changes are observed in the salivary gland during and after blood-feeding, and apoptosis occurs in the process of salivary gland degeneration [13, 14]. However, the molecular mechanisms involved in the regulation of this process have not been directly established.

Salivary gland degeneration after engorgement in the adult female tick *Amblyomma hebraeum* is triggered by an ecdysteroid hormone. This degeneration, for both in vivo and in vitro conditions, requires 4 days to complete [15, 16]. In addition, ecdysteroids such as 20-hydroxyecdysone (20E) can cause the degeneration of the salivary gland when infused into the tick hemocoel or when the salivary gland is exposed, in vitro, to physiological amounts of the ecdysteroid [16]. Programmed cell death can occur within 48–72 h post-engorgement of ticks [13, 17]. These data suggest that the degeneration of the salivary gland is regulated, at least in part, by steroid hormones. Ecdysteroids are steroid hormones that control differentiation, reproduction, homeostasis, and other biological processes in vertebrates and invertebrates. In arthropods, ecdysteroid hormones, including 20E, are the primary steroid hormones and are essential for successful molting and metamorphosis. Ecdysteroid hormones influence arthropod development and reproduction by binding a heterodimeric complex of nuclear receptors, including the ecdysone receptor (EcR) and ultraspiracle (USP) [18]. The molecular mechanism of the ecdysteroid receptor is relatively well known in insects, but less is known about the molecular mechanisms of ecdysteroid action in ticks**.**

Argasid and ixodid ticks both contain ecdysone and 20E and accumulate a variety of metabolites, similar to insects [19]. Ecdysteroids have been identified in all life stages of ticks, and they influence molting, diapause, spermatogenesis [16], and salivary gland degeneration [15, 20]. The tick ecdysteroid receptor reversibly binds to the ecdysone analog ponasterone A (PonA). Saturable and reversible ligation was observed in crude salivary gland extracts of partially fed *A. hebraeum* [21]. In contrast to the biochemistry and molecular mechanisms of ecdysone in insects, relatively little is known about ecdysteroid biosynthesis, metabolism, or mode of action in ticks.

Steroids perform their biological functions on target cells predominantly through the binding and activation of their nuclear hormone receptors [22]. In insects, the functional ecdysone receptor is a heterodimer composed of two proteins, the ecdysone receptor (EcR: NR1H1) and ultraspiracle (USP: NR2B4) [18, 23, 24]. The EcR is found only in arthropods, and USP is the invertebrate homolog to the mammalian retinoic X receptor (RXR), which forms promiscuous heterodimers with several nuclear hormone receptors in vertebrates. These molecules have been isolated from several insect orders and from other arthropods. Both EcR and USP are members of the nuclear hormone superfamily that includes proteins which are critical for normal development and metabolism, mediated by genomic ecdysone effects [25]. However, in a manner analogous to vertebrates, EcR and USP also have extranuclear actions that participate in non-genomic ecdysone effects [15, 24, 26–28]. Both EcR and USP have multiple isoforms that differ only in their N-terminal A/B domain. They have the same highly conserved C domain (DNA-binding domain, DBD), D domain (hinge), and conserved E domain (ligand-binding domain, LBD). In A. hebraeum ticks, three isoforms of EcR, namely AamEcR1, AamEcR2, and AamEcR3, have been isolated [29]. Two USP isoforms in A. hebraeum, AamRXR1 and AamRXR2, have been identified [30]. These receptors induce the apoptotic process, a programmed cell death with the cleavage of caspases as well as several other proteins.

In female ticks, ecdysteroids regulate salivary gland degeneration [16]. Apoptosis occurs during salivary gland degeneration [31], but the molecular mechanisms involved in the apoptosis-related process are unknown. The goal of this study was to identify the ecdysteroid receptor and to characterize its role during blood-feeding and in the degeneration of the *Rhipicephalus haemaphysaloides* salivary gland by regulating caspase-dependent apoptosis.

## Methods

### Rearing of ticks

All stages of ticks (*R. haemaphysaloides*, a cattle tick from Wuhan, China) were reared in the laboratory on the ears of New Zealand white rabbits at Shanghai Veterinary Research Institute, Chinese Academy of Agricultural Sciences. Ticks were kept in a dark incubator at 27 °C and 95% humidity in the laboratory as described previously [1]. Adult males and females were fed together to permit mating on the host.

### Collection of hemolymph and dissection of tissues from ticks

Ticks were cleaned in water, dried, and glued (cyanoacrylate compound) to the bottom of a transparent Petri dish, and placed under crushed ice for 15 min. Afterward, the ice was removed, and a small incision was made along the dorsolateral margin. Exuding hemolymph (up to 30 μl) was collected in capillary tubes, and 300 μl methanol was added immediately. It was then frozen quickly by liquid nitrogen immersion and stored at −80 °C until 20E extraction and detection.

For tissue collection, after identical tick preparation, a small incision was made along the dorsolateral margin, and the dorsal surface was removed with a scalpel blade. The coelom was washed with cooled phosphate-buffered saline (PBS) and the salivary gland, fat body, gut, ovary, and synganglion were removed. These organs were then immediately separated with fine-tipped forceps under a light microscope [1]. The sample materials were quickly frozen in liquid nitrogen and stored at −80 °C until use (for RNA and protein analysis). A section of the salivary gland (10 mg) was homogenized using an ultrasonic instrument, and 300 μl methanol was added immediately; it was then frozen quickly by liquid nitrogen immersion and stored at −80 °C until 20E extraction and detection.

### Cloning the open reading frame (ORF) of the *RhEcR* and *RhUSP* genes

According to the salivary gland transcriptomes of engorged *R. haemaphysaloides* in our laboratory, we localized ORF regions of *RhEcR* and *RhUSP* in contig sequences (Additional file 2). Primers (Additional file 1: Table S1) were designed to clone these sequences. The amplicons with complete ORF sequences were cloned into the pET-28a vector using the ClonExpress II One Step Cloning Kit (Vazyme Biotech, Nanjing, China), and the obtained clones were sequenced. Signal peptide analysis, isoelectric point/molecular weight (PI/Mw), and nuclear localization signal (NLS)/nuclear export signal (NES) were predicted by the online software SignalP 5.0 (http://www.cbs.dtu.dk/services/SignalP/) [32], ExPASy (https://web.expasy.org/compute_pi/) [33], and NLS/NES signal (https://rostlab.org/services/nlsdb/) [34]. Similar sequences of target genes were searched using the Smart BLAST (or Protein BLAST) server (National Center for Biotechnology Information, National Institutes of Health). The sequences of RhEcR1, 2, and 3 and RhUSP1 and 3 were aligned with the EcR/LXR and the USP/RXR of other species using Genetyx version 6 software (Genetyx, Tokyo, Japan). For phylogenetic analysis, the alignment of the sequences was performed using the CLUSTALW algorithm [35] and inferred using the maximum likelihood method with default settings in MEGA 6.0 software [36]. The sequences of EcR/LXR and USP/RXR in vertebrates and invertebrates were obtained from the GenBank database (Additional file 1: Table S2).

### Expression analysis of genes by quantitative real-time polymerase chain reaction (qRT-PCR)

The *RhEcR* and *RhUSP* gene expression profiles in the salivary gland, gut, fat body, ovary, and synganglion from adult female ticks who were unfed, after feeding for 3 days (Fed 3) and 5 days (Fed 5), and post-engorgement at 0 h (E 0h), 24 h (E 24h), 48 h (E 48h), 72 h (E 72h), and 96 h (E 96h) were determined by qRT-PCR using the 2^−△CT^ analytical method. Ten female ticks per condition were analyzed. The data were normalized to elongation factor 1-alpha (EF1α). Total RNA was extracted using TRIzol reagent (15-596-018, Invitrogen, USA) and transcribed to synthesize complementary DNA (cDNA) using HiScript^®^ III RT SuperMix (R323, Vazyme Biotech, Nanjing, China), using the following program: 42 °C for 2 min, 37 °C for 15 min, 85 °C for 5 s, and finally 16 °C for the synthesis of cDNA. The cDNA samples were analyzed by qRT-PCR using the ChamQ Universal SYBR qPCR Master Mix (Q711, Vazyme Biotech, Nanjing, China) with a QuantStudio™ 5 Real-Time PCR System (Applied Biosystems™, Waltham, MA, USA), with program parameters of 95 °C for 30 s; 40 cycles of 95 °C for 10 s and 60 °C for 30 s; and 95 °C for 15 s, 60 °C for 60 s, and 95 °C for 15 s.

### Preparation of polyclonal antibodies (PcAb) in mice

The epitopes of RhEcR were analyzed by antibody epitope prediction using BepiPred Linear Epitope Prediction 2.0 (http://tools.iedb.org/bcell/) [37]. The peptide sequences were selected and synthesized (GL Biochem, China) and coupled to the keyhole limpet hemocyanin (KLH) carrier protein. Antibodies against the peptides were raised in BALB/C mice by intraperitoneal immunization with 100 μg of the peptide in PBS emulsified with the same volume of Freund’s complete adjuvant (Sigma-Aldrich, St. Louis, MO, USA). Eight mice in each group, which were maintained in a specific-pathogen-free (SPF) animal area, were inoculated three times at 15-day intervals with 50 μg of peptide in PBS emulsified with the same volume of Freund’s incomplete adjuvant (Sigma-Aldrich, St. Louis, MO, USA). Seven days after the final inoculation, sera were collected and stored at −20 °C until use.

### 20-Hydroxyecdysone measurement using enzyme immunoassay (EIA)

The 20E levels in the salivary gland and the hemolymph were measured using an enzyme immunoassay (EIA) according to the manufacturer’s instructions (20-Hydroxyecdysone ELISA kit, #A05120.96 wells, Bertin, France). From female ticks of different feeding periods, we collected 10 mg of the salivary glands by dissection and 30 μl of hemolymph by capillary absorption during each time course.

### Western blot

Total protein was extracted from the salivary gland at each time point in RIPA lysis buffer containing protease inhibitor, with 1 mM phenylmethanesulfonyl fluoride (PMSF), followed by centrifugation at 16,000×*g* at 4 °C for 15 min. The supernatant was collected, and a BCA protein assay kit (Thermo Fisher Scientific, Waltham, MA, USA) was used to determine the protein concentration following the manufacturer’s instructions. For sodium dodecyl sulfate–polyacrylamide gel electrophoresis (SDS-PAGE) and western blot analysis, 20–50 µg of total protein per sample was applied to 10% SDS-PAGE and transferred to a polyvinylidene fluoride (PVDF) membrane. The PVDF membrane was incubated with antibodies at 4 °C overnight and then with goat anti-mouse or goat anti-rabbit conjugated with horseradish peroxidase (HRP; D20691 or A32731, Invitrogen, USA) at room temperature for 2 h. Images were captured using the ChemiDoc Touch Imaging System (Bio-Rad, Hercules, CA, USA).

### RNA interference for gene knockdown in ticks

#### Synthesis of double-stranded RNA

Double-stranded RNA (dsRNA) was synthesized using the T7 RiboMAX™ RNAi System (P1700, Promega, Madison, WI, USA) according to manufacturer’s recommendations. Primers containing T7 promoter sequences (Additional file 1: Table S1) were designed to synthesize the dsRNA. The dsRNA to *RhEcR*, *RhUSP*, and *Luciferase* mRNA had about 500 base pairs (bp) each. Because the *R. haemaphysaloides* genome sequence is not available, the siRNA specificity and potential off-targets were estimated based on similar regions of the *Drosophila melanogaster* ortholog gene (accession number XP_021710216) and genome sequences using the dsCheck program [38]. Also, using BLAST, analyses were conducted using publicly available genome sequences of *Ixodes persulcatus*, *Haemaphysalis longicornis*, *Dermacentor silvarum*, *Hyalomma asiaticum*, *R. sanguineus*, *R. microplus*, and *I. scapularis* to confirm that the dsRNA was specific to the target genes and to reduce the possibility of off-target effects. A dsRNA targeting luciferase was used as a negative control [39]. The quality of the dsRNA was determined by electrophoresis on 1.0% agarose gel. The dsRNA was stored at −80 °C until use.

### RNAi experiment

Virgin or unfed adult female ticks (*n* = 80 females per group, two independent groups) were micro-injected with approximately 0.5 μl dsRNA (containing about 1 μg) to *RhEcR*, *RhUSP*, and *Luciferase* at the base of the fourth right leg (near the genital aperture) of the ventral surface. After microinjection, ticks were maintained in a dark incubator at 27 °C with 95% humidity for 24 h, and they were then allowed to feed on rabbit ears. Four female ticks per group were collected after 5 days of feeding for RNA extraction using TRIzol reagent (15–596-018, Invitrogen, USA), following the manufacturer’s instructions. qRT-PCR was performed to characterize gene knockdown efficiency using the 2 ^−△△CT^ analytical method. Ten female ticks per group were analyzed. The data were normalized to EF1α. The remaining ticks were allowed to feed until they detached. The tick 24-h attachment rate, engorgement rate, engorgement weight, oviposition rate, and egg-hatching rate were determined for the individual female ticks.

### Histology and HE staining

The salivary glands of each groups were removed from adult female ticks by micro-dissection at different time points during blood-feeding and after engorgement. Salivary glands were fixed in 4% paraformaldehyde and embedded with paraffin. Paraffin sections were stained with hematoxylin–eosin (HE). HE staining images were obtained by light microscopy (Nikon Eclipse 80i microscopy system using a ×40 objective).

### Quantitative proteomic analysis by tandem mass tag (TMT) technology

The salivary glands of normal female ticks (Fed 3, Fed 5, E 0h, and E 72h) and dsEcR-treated/dsLuciferase-treated ticks (Fed 3, Fed 5, and Fed 7) were dissected and washed with ice-cold PBS, then frozen quickly by liquid nitrogen immersion and stored at −80 °C. Each sample had 15–20 female ticks and two repeats. All the samples were treated and tagged with TMT and analyzed by Shanghai Applied Protein Technology.

### Co-transfection and co-immunoprecipitation

HEK293T cells were seeded onto a six-well plate. Cells with 70–80% confluence were co-transfected with the plasmids pCMV-Flag-RhEcR1, pCMV-Flag-RhEcR2, pCMV-Flag-RhEcR3, pCMV-HA-RhUSP1, pCMV-HA-RhUSP3, GFP-RhEcR1, and mCherry-RhUSP1 using Lipofectamine™ 3000 reagent according to the manufacturer’s instructions (L3000008, Invitrogen, USA). After 24 h, the cellular supernatant was collected to detect the cytotoxicity for detection of cell death using the ToxiLight™ bioassay kit according to manufacturer’s instructions (LT07-117, Lonza, Basel, Switzerland). The transfected cells were collected and protein was extracted using a lysis buffer (50 mM Tris, 150 mM NaCl, 0.5% NP-40 (v/v), pH 7.5) containing protease inhibitor for the immunoprecipitation assay. Cell extract supernatants were incubated with anti-Flag immunomagnetic beads (B26101, Bimake, USA) and anti-HA immunomagnetic beads (B26201, Bimake, USA) according to the manufacturer’s instructions for 7–8 h at 4 °C with gentle shaking. The protein–antibody complex with immunomagnetic beads was separated magnetically and washed three times with PBST. After adding 50 μl 1× protein loading buffer and boiling for 5 min, the samples were cooled to room temperature and placed on a magnetic rack for 10 s. Finally, the protein supernatant was used for the SDS-PAGE test. The samples were subjected to SDS/PAGE (10% gel, GenScript, Nanjing, China), followed by western blot analysis with rabbit monoclonal antibodies against Flag (14793S, Cell Signaling Technology, Danvers, MA, USA) and HA (3724S, Cell Signaling Technology) to detect the target proteins. GFP-EcR1 and mCherry-USP1 were co-transfected into HEK293T cells for 24 h to detect the expression of proteins under a fluorescence microscope. We then discarded the supernatant, fixed the cells, stained the cells with DAPI for 5–10 min, and observed proteins under a fluorescence microscope.

### Data analysis

All statistical analyses were performed using GraphPad Prism 6.0 software (GraphPad Software Inc., San Diego, CA, USA). One-way and two-way analysis of variance (ANOVA) for statistical differences between multiple groups was used (α = 0.05). Mean ± standard deviation (SD) values were calculated for three separate experiments, and two-tailed Student’s *t*-tests were used to identify significant differences (*P < 0.05, **P < 0.01, ***P < 0.001, ****P < 0.0001) between groups.

## Results

### Identification of ecdysteroid receptor in the salivary gland of *R. haemaphysaloides*

**Fig. 1:**
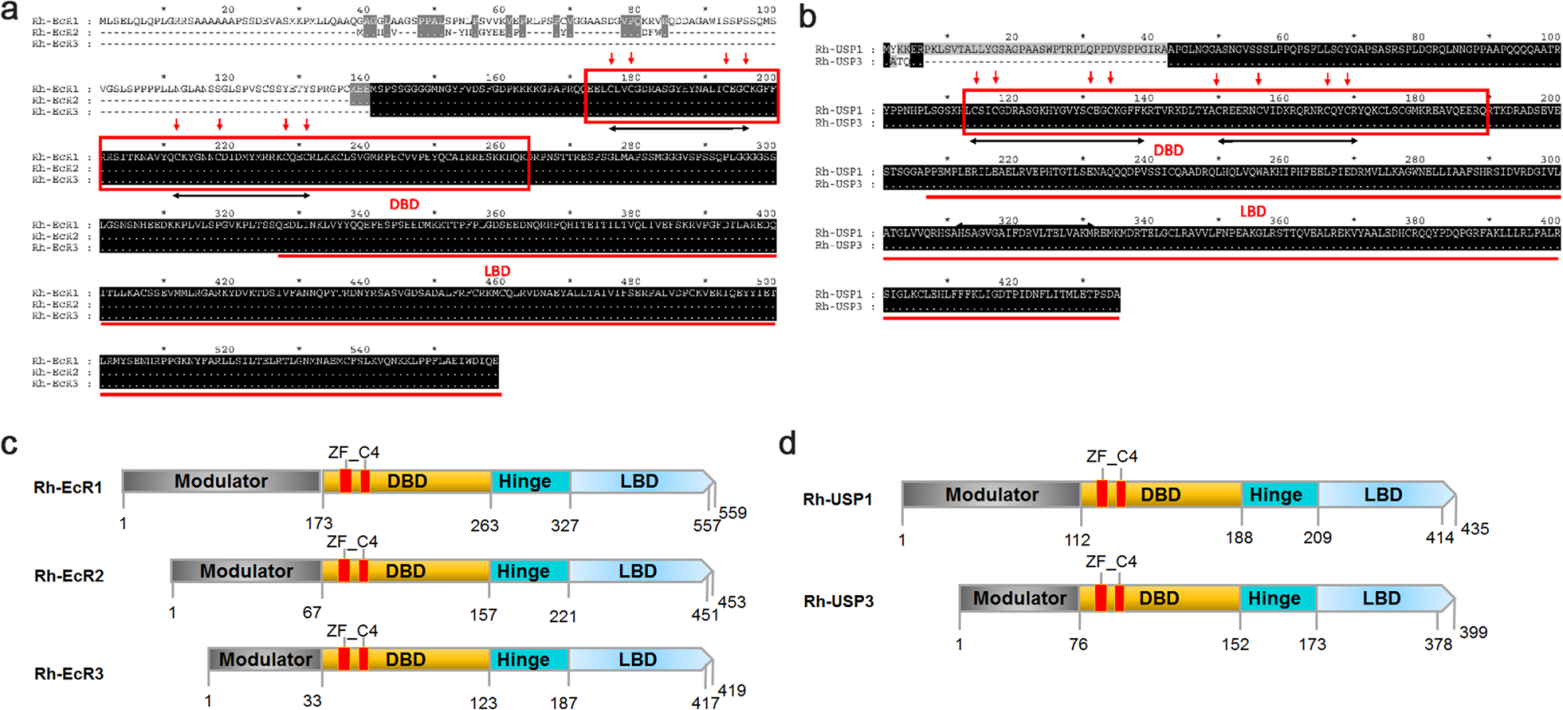
The cloning of the ecdysteroid receptor heterodimeric complex (EcR and USP) of *Rhipicephalus haemaphysaloides.* The ORFs of *RhEcR1*, *RhEcR2*, *RhEcR3*, *RhUSP1*, and *RhUSP3* from *R. haemaphysaloides* were cloned, sequenced, and characterized in silico, and designated (**a**, **b**, **c**, and **d**). Nucleotide and deduced amino acid sequences of *RhEcR1*, *RhEcR2*, *RhEcR3*, *RhUSP1*, and *RhUSP3* from *R. haemaphysaloides* (**a** and **b**). Putative DNA-binding domain (DBD) is boxed and ligand-binding domain (LBD) is underlined. Two C4-type zinc-finger motifs (ZF_C4) have DNA-binding regions of some well-characterized families of nuclear receptors, and each ZF_C4 is characterized by four cysteine residues, which are marked with arrows, that coordinate zinc and do not share sequence similarity. The distances between the cysteine residues are not conserved. The domain structure is drawn for RhEcR1, 2, and 3 and RhUSP1 and 3 (**c** and **d**)

A salivary gland transcriptome produced by our laboratory was used to identify the open reading frame (ORF) sequences of the three isoforms of *RhEcR* and the two isoforms of *RhUSP* (three contigs for *RhEcR*: CL2185_contig1, contig2, and contig4 and one contig for *RhUSP*). The sequences have typical structural characteristics of nuclear receptors including a variable N-terminal domain (A/B), a highly conserved DBD (C), a variable hinge region (D), and a moderately conserved LBD (E). Subsequently, the ORF regions of the three *RhEcR* isoforms and two *RhUSP* isoforms were cloned from the cDNA of the salivary gland from fully engorged female *R. haemaphysaloides* and named *RhEcR1*, *RhEcR2*, *RhEcR3*, *RhUSP1*, and *RhUSP3*, respectively. The ORFs of *RhEcR1*, *RhEcR2*, and *RhEcR3* have 1680 bp, 1362 bp, and 1260 bp, with deduced molecular weights (MW) and theoretical isoelectric points (PI) of 61.3 kDa /8.39, 51.2 kDa /7.80, and 47.2 kDa/8.48, respectively. The ORFs of *RhUSP1* and *RhUSP3* have 1308 bp and 1200 bp, with deduced molecular weights (MW) and theoretical isoelectric points (PI) of 48.2 kDa/8.93 and 44.4 kDa/8.60, respectively. RhEcR has sequence similarity to the EcR of various insects, other arthropods, and the LXR of mammals (Additional file 1: Figure S1). RhUSP also has sequence similarity to the USP of insects, arthropods, and the RXR of mammals (Additional file 1: Figure S2). *RhEcR* and *RhUSP* have a conserved DBD that has two conserved ZF_C4 motifs characterized by four cysteine residues and one conserved LBD (Fig. 1a and b). Similar to other nuclear receptors, they also have one variable N-terminal A/B domain, a highly conserved DBD (C), a variable hinge region (D), one variable hinge region (E), and a moderately conserved LBD, but not the variable C-terminal region (Fig. 1c and d). The sequence alignments showed that the DBD of arthropod EcR are conserved with mammalian LXR and have two ZF_C4 motifs (Additional file 1: Figure S1). Similarly, the DBD of arthropod USP and mammalian RXR also have two conserved ZF_C4 motifs (Additional file 1: Figure S2). Phylogenetic analysis (Additional file 1: Figure S3) showed that the RhEcR and RhUSP sequences in this study were more closely related to the sequences of other ticks than to mammalian homologous sequences. Furthermore, a BLAST search against genomic sequences of six tick species identified only a sequence of each RhEcR and RhUSP sequences (Additional file 1: Figure S4). These data indicate that the *EcR* and *USP* receptor gene homologs are present in *R. haemaphysaloides*.

### Tissue specificity and temporal dynamics of *RhEcR* and *RhUSP* expression during the blood-feeding process and post-engorgement

**Fig. 2:**
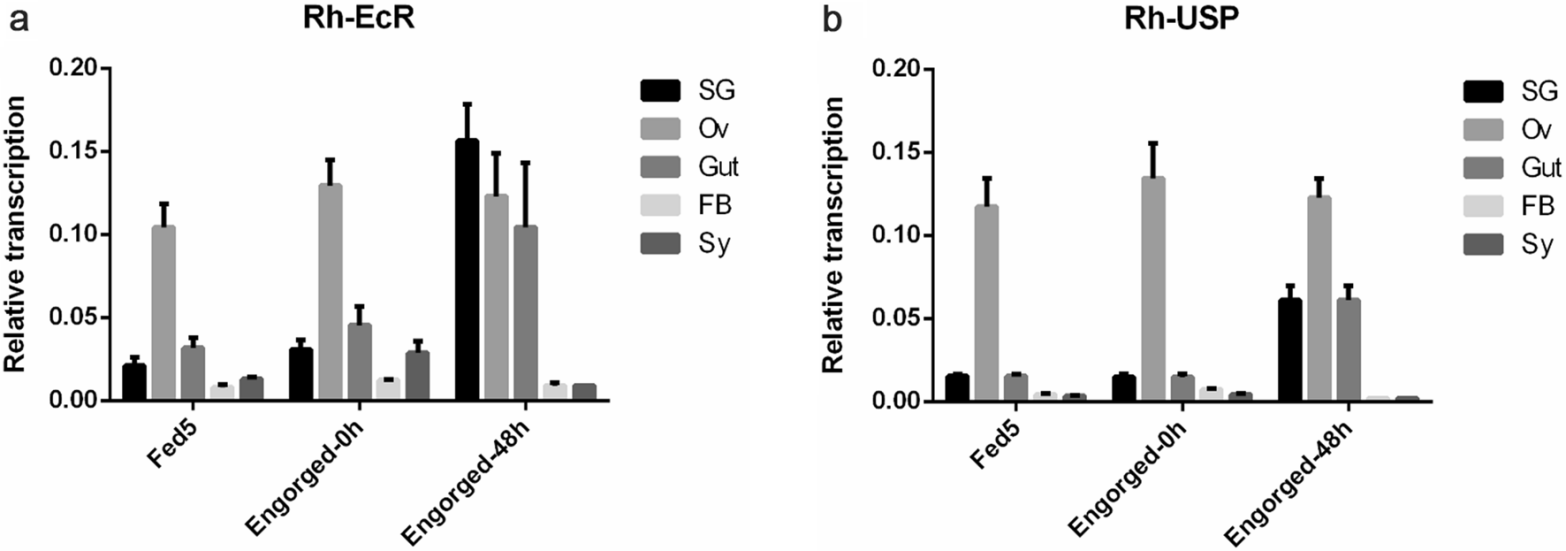
Tissue and temporal distribution of ecdysteroid receptor (*EcR* and *USP)* of *Rhipicephalus haemaphysaloides.* Tissues were dissected from female ticks. Using qRT-PCR, relative RNA levels of *RhEcR* and *RhUSP* were measured at each time point. Temporal distribution profile of **a** *RhEcR* and **b** *RhUSP* in the salivary gland, fat body, gut, ovary, and synganglion during the blood-feeding and post-engorgement periods. Both *RhEcR* and *RhUSP* showed a wide time and tissue distribution profile. The data represent the mean ± SD of the experiment (10 ticks/time point) performed in triplicate and normalized to elongation factor 1-alpha (EF1α)

Using qRT-PCR, the tissue-specific and temporal distribution profiles of the transcripts of the components of the ecdysone receptor complex, *RhEcR* and *RhUSP*, were determined. The tissue distribution profile was studied in the salivary gland (SG), fat body (FB), ovary (Ov), and synganglion (Sy) of blood-feeding and post-engorged adult female ticks. The temporal distribution profile was performed on the third and fifth days during the blood-feeding period (Fed 3 and Fed 5) and after engorgement (E 48h). Both components of the ecdysone receptor complex, *RhEcR* and *RhUSP*, showed a wide time and tissue distribution profile (Fig. 2a and b). In the SG, both *RhEcR* and *RhUSP* had a higher RNA level at E 24h, from which the SG underwent a higher degree of degeneration (Additional file 1: Figure S5a and b). In the Ov, when the weight of female ticks reached the “critical weight” (the beginning of the fast blood-feeding period, around 5–6 days), *RhEcR* and *RhUSP* RNA levels increased rapidly, and this high level was maintained until the beginning of oviposition (Additional file 1: Figure S5c and d). In the Gut, levels of *RhEcR* and *RhUSP* RNA were higher after E 24h, and the Gut also underwent degeneration (Additional file 1: Figure S5g and h). In FB, *RhEcR* RNA levels increased from Fed 5 until E 24h and then decreased thereafter, except at E 96h (Additional file 1: Figure S5e); *RhUSP* RNA levels increased from Fed 5 until E 0h and then decreased until E 72h, while RNA levels at E 96h were substantially increased (Additional file 1: Figure S5f). However, in Sy, *RhEcR* RNA levels increased from Fed 3 until E 24h and decreased thereafter (Additional file 1: Figure S5i); for the *RhUSP* gene, however, the RNA levels were lower, so it was difficult to observe these variations (Additional file 1: Figure S5j).

**Fig. 3:**
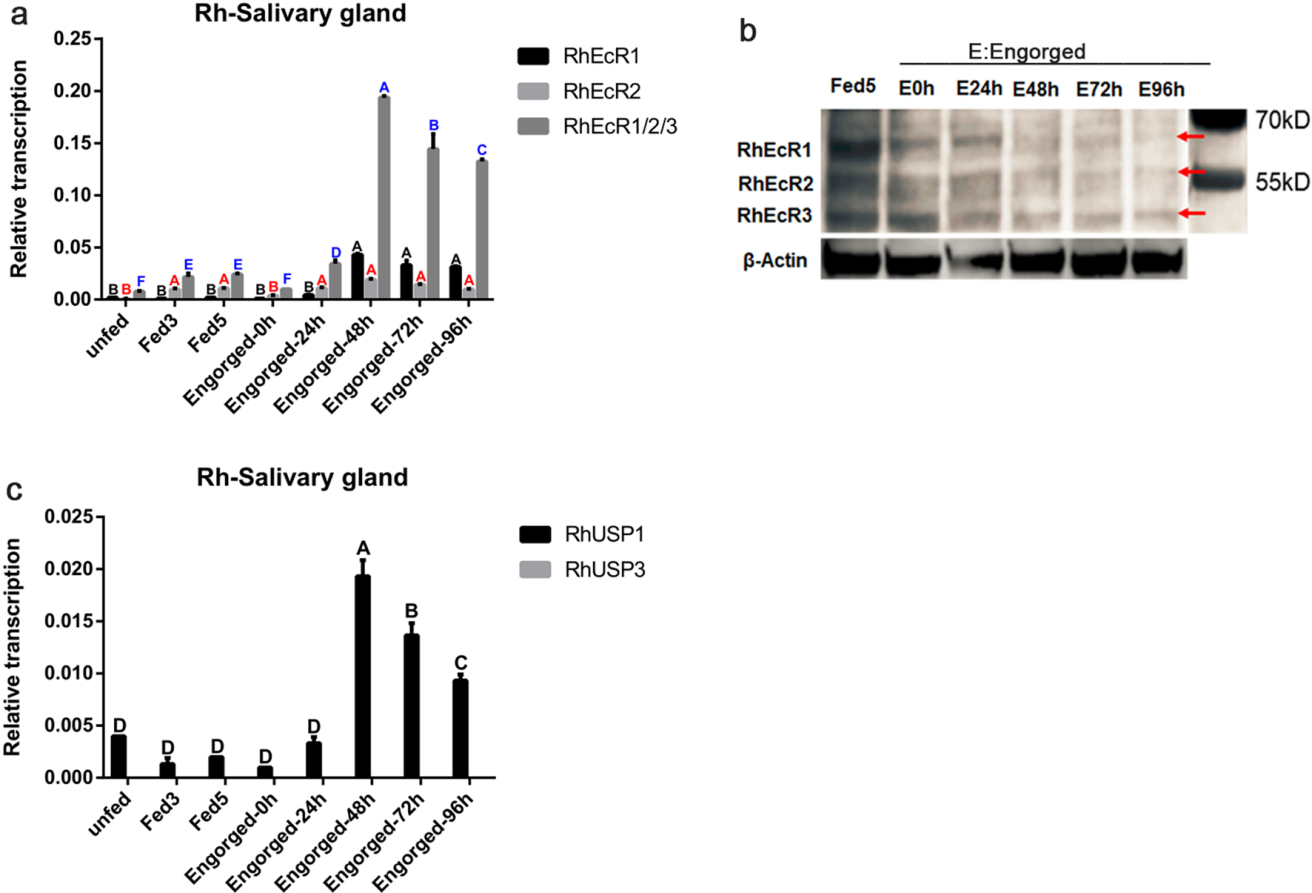
Temporal distribution of RhEcR and RhUSP isoforms in the salivary gland. Tissues were dissected from female ticks. Using qRT-PCR, relative RNA levels of *RhEcR* and *RhUSP* were measured at each time point. **a** In the period of blood-feeding, the expression of *RhEcR2* was higher than *RhEcR1* before E 24 h. After E 24 h, the expression of *RhEcR1* was higher than *RhEcR2* (ANOVA, RhEcR1/2/3, Engorged-48h vs Engorged-72h, *F* (2, 24), *P* < 0.0001; Engorged-72h vs Engorged-96h, *F* (2, 24), *P* = 0.0012; Engorged-96h vs Engorged-24h, *F* (2, 24), *P* < 0.0001; Engorged-24h vs Fed5, *F* (2, 24), *P* = 0.008; Fed5 vs Fed3, *F* (2, 24), *P* = 0.9877; Fed3 vs Engorged-0h, *F* (2, 24), *P* = 0.0018; Engorged-0h vs unfed, *F* (2, 24), *P* = 0.9877; RhEcR1, Engorged-48h vs Engorged-72h, *F* (2, 24), *P* = 0.1115; Engorged-72h vs Engorged-96h, *F* (2, 24), *P* = 0.9984; Engorged-96h vs Engorged-24h, *F* (2, 24), *P* < 0.0001; Engorged-24h vs Fed5, *F* (2, 24), *P* = 0.9877; Fed5 vs Fed3, *F* (2, 24), *P* > 0.9999; Fed3 vs Engorged-0h, *F* (2, 24), *P* > 0.9999; Engorged-0h vs unfed, *F* (2, 24), *P* = 0.9877; RhEcR2, Engorged-48h vs Engorged-72h, *F* (2, 24), *P* = 0.6662; Engorged-72h vs Engorged-96h, *F* (2, 24), *P* = 0.6662; Engorged-96h vs Engorged-24h, *F* (2, 24), *P* > 0.9999; Engorged-24h vs Fed5, *F* (2, 24), *P* > 0.9999; Fed5 vs Fed3, *F* (2, 24), *P* > 0.9999; Fed3 vs Engorged-0h, *F* (2, 24), *P* = 0.023; Engorged-0h vs unfed, *F* (2, 24), *P* = 0.9877). **b** The translation of RhEcR isoforms was detected with anti-mouse RhEcR polyclonal antibody. After E 24 h, the expression of all isoforms of RhEcR gradually decreased. **c** Two isoforms of *RhUSP* were detected during all time points, showing that the RNA levels of *RhUSP1* were high and *RhUSP3* were very low (ANOVA, RhUSP1, Engorged-48h vs Engorged-72h, *F* (1, 17), *P* < 0.0001; Engorged-72h vs Engorged-96h, *F* (1, 17), *P* < 0.0001; Engorged-96h vs unfed, *F* (1, 17), *P* < 0.0001; unfed vs Engorged-24h, *F* (1, 17), *P* = 0.9855; Engorged-24h vs Fed5, *F* (1, 17), *P* = 0.1282; Fed5 vs Fed3, *F* (1, 17), *P* = 0.9855; Fed3 vs Engorged-0h, *F* (1, 17), *P* > 0.9999). The data represent the mean ± SD of the experiment (10 ticks/time point) performed in triplicate and normalized to EF1α, and 2 way ANOVA multiple comparisions for statistical differences between multiple groups were used (α = 0.05)

To refine the expression analysis, salivary glands of the blood-feeding and post-engorgement female ticks were used to identify and quantify individual isoform expression of the three isoforms of RhEcR and two isoforms of RhUSP. During the period of blood-feeding and until E 24h, the *RhEcR2* RNA level was higher than that of *RhEcR1*, after which the *RhEcR1* RNA level was higher than that of *RhEcR2* (Fig. 3a). Western blot analysis using anti-RhEcR serum showed that the protein level of RhEcR1 in the salivary gland was increased on the fifth day of the feeding period and decreased from E 48h. *RhEcR2* was already expressed during the Fed 5 period and remained stable until E 0h. It then gradually decreased after E 0h (Fig. 3b). In comparison, the *RhUSP1* RNA level increased before 48 h post-engorgement and decreased thereafter until the end of the experiment. In contrast, *RhUSP3* RNA levels remained low at all the time points (Fig. 3c).

### Dynamic concentration of 20E during tick blood-feeding and post-engorgement periods

**Fig. 4:**
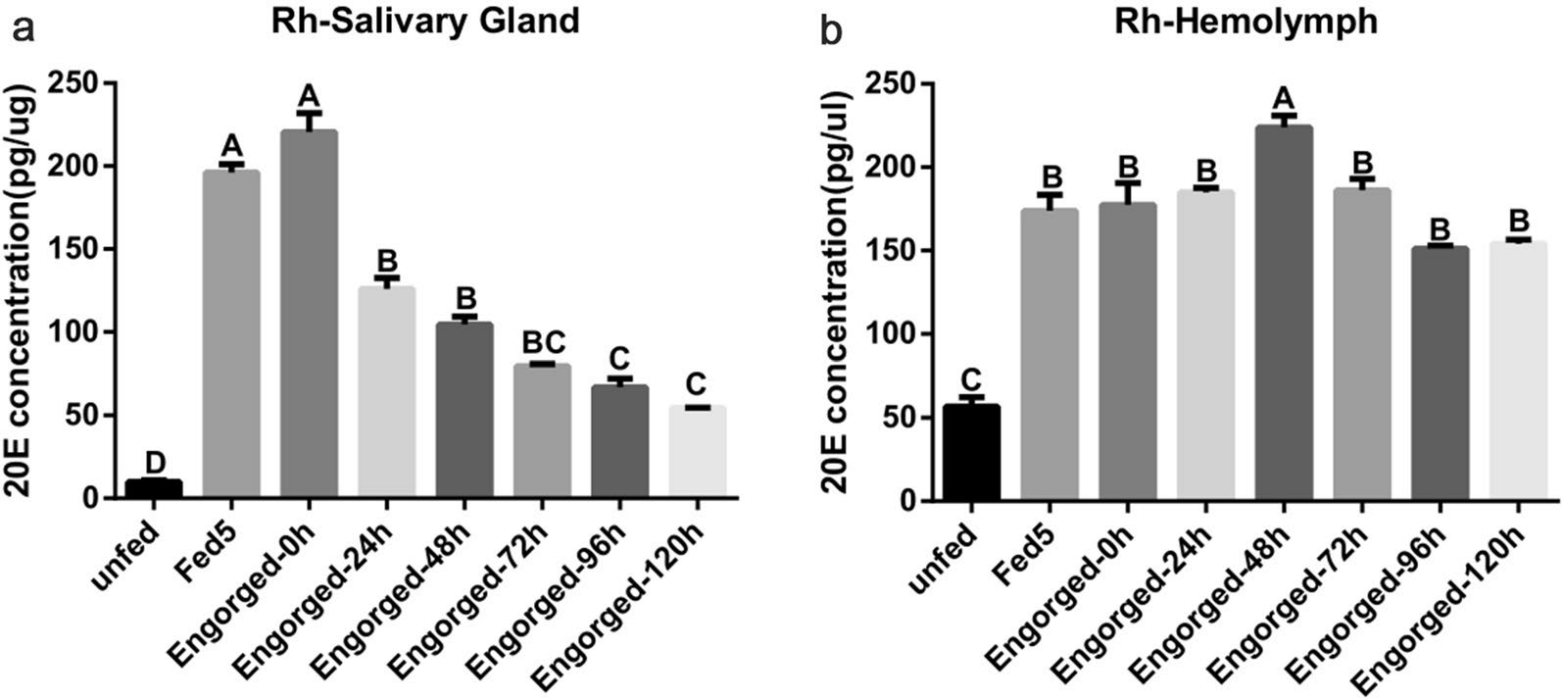
The 20E concentration in the salivary gland and hemolymph of *Rhipicephalus haemaphysaloides*. Tissues were dissected from female ticks. The 20E concentration in **a** the salivary gland (ANOVA, Engorged-0h vs Fed5, *F* (7, 8), *P* = 0.9994; Fed5 vs Engorged-24h, *F* (7, 8), *P* < 0.0001; Engorged-24h vs Engorged-48h, *F* (7, 8), *P* = 0.0619; Engorged-48h vs Engorged-72h, *F* (7, 8), *P* = 0.0289; Engorged-72h vs Engorged-96h, *F* (7, 8), *P* = 0.424; Engorged-96h vs Engorged-120h, *F* (7, 8), *P* = 0.4278; Engorged-120h vs unfed, *F* (7, 8), *P* = 0.0007) and **b** hemolymph (ANOVA, Engorged-48h vs Engorged-72h, *F* (7, 8), *P* = 0.0101; Engorged-72h vs Engorged-24h, *F* (7, 8), *P* > 0.9999; Engorged-24h vs Engorged-0h, *F* (7, 8), *P* = 0.9455; Engorged-0h vs Fed5, *F* (7, 8), *P* = 0.9994; Fed5 vs Engorged-120h, *F* (7, 8), *P* = 0.2216; Engorged-120h vs Engorged-96h, *F* (7, 8), *P* = 0.9998; Engorged-96h vs unfed, *F* (7, 8), *P* < 0.0001), during blood-feeding and the post-engorgement period were measured by EIA (in pg/μg or μl tissue). The data represent the mean ± SD of with 10 mg salivary gland or 30 μl hemolymph at each time point, and samples from different ticks were analyzed in duplicate. One-way ANOVA for statistical differences between multiple groups was used (α = 0.05)

Temporal changes in 20E concentration in the salivary gland and hemolymph were analyzed using an enzyme immunoassay. In the salivary gland of adult female ticks, 20E concentration was lower in the unfed stage, increased between Fed 5 and engorgement (the peak of 20E concentration in the salivary gland), and decreased afterward (Fig. 4a). In the hemolymph, the 20E concentration was lower in unfed ticks, increased until Fed 5, and remained stable thereafter (Fig. 4b).

### *RhEcR* gene silencing blocked tick blood-feeding

#### Knockdown efficiency

**Fig. 5:**
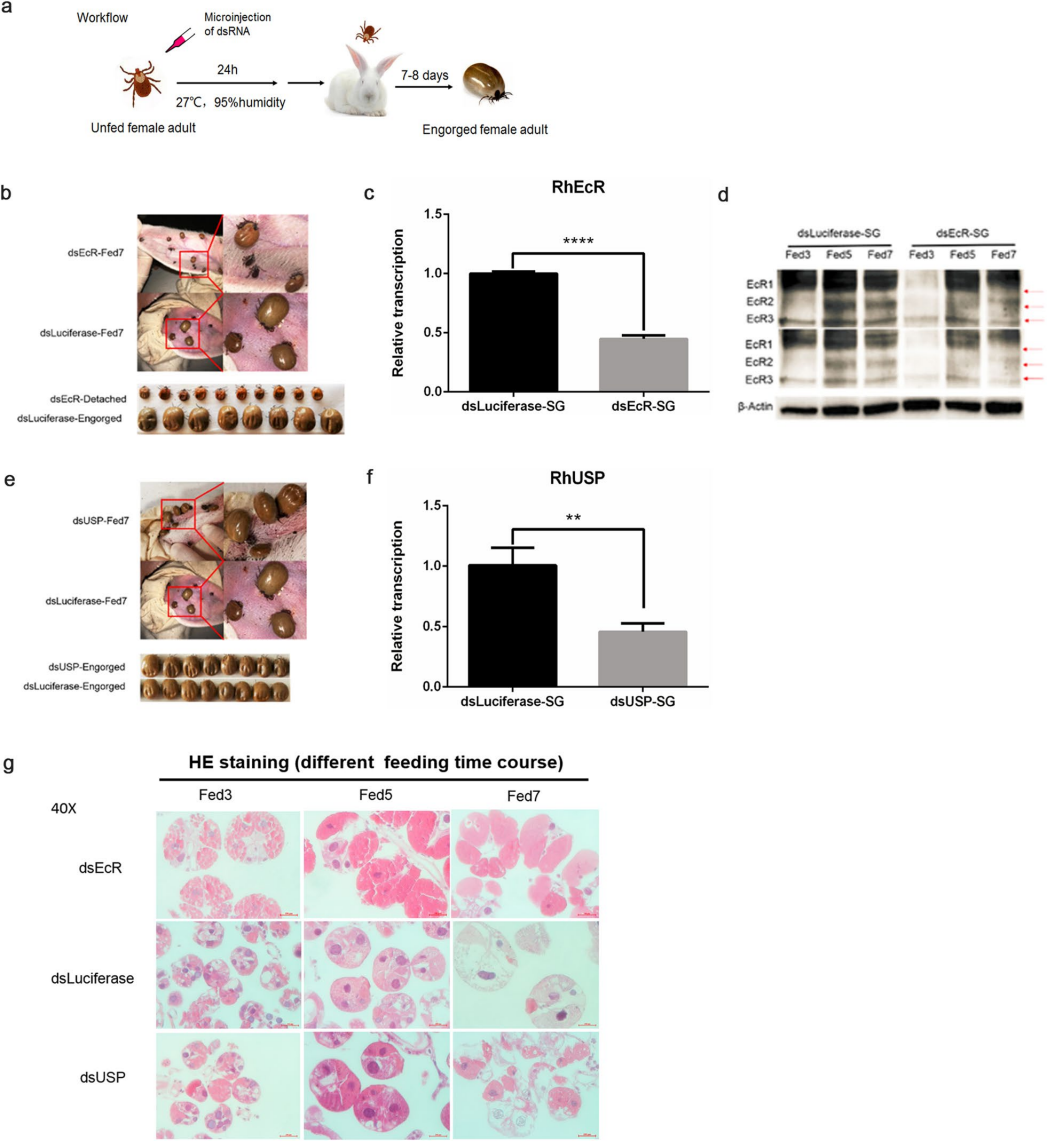
Biological effects on dsRhEcR- or dsRhUSP-treated ticks. Female ticks were treated with dsRhEcR or dsRhUSP. The knockdown of *RhEcR* affected the normal growth and development of ticks and the salivary gland degeneration**.** **a** Schematic workflow of the RNAi experiments. **b** DsRhEcR treatment inhibited blood-feeding and growth compared to the dsLuciferase-treated group. **c** *RhEcR* was significantly reduced compared to the dsLuciferase-treated group (t = 28.91, df = 4, *P* < 0.0001). **d** The dsRhEcR-treated group also showed reduced RhEcR protein levels in the salivary gland compared to the dsLuciferase-treated group. **e** DsRhUSP treatment had no significant effect on blood-feeding or growth compared to the dsLuciferase-treated group. **f** The DsRhUSP-treated group showed no morphological alterations compared to the dsLuciferase-treated group, although *RhUSP* was significantly reduced compared to the dsLuciferase-treated group (t = 5.885, df = 4, *P* = 0.0042). **g** HE staining of the salivary gland within the blood-feeding period in dsRhEcR-, dsRhUSP- and dsLuciferase-treated groups. The agranular acini morphology in dsRhEcR-treated ticks was abnormal, since it showed increased size of the acini. The most obvious effect was that agranular acini did not show the degenerated morphology. In **c** and **f**, the data represent the mean ± SD of the experiment (10 ticks/time point) performed in triplicate and normalized to EF1α, and two-tailed Student’s *t*-tests were used to identify significant differences between groups (****P < 0.0001, **P < 0.01)

A well-established workflow based on RNA interference (RNAi) was used to inhibit RhEcR/RhUSP expression and to analyze its effect on the morphology of adult female ticks (Fig. 5a). RNAi-mediated knockdown of *RhEcR* resulted in a significant reduction in both the RNA and protein levels of RhEcR in the salivary gland (Fig. 5c, d and f). These results showed successful gene silencing by RNAi of *RhEcR* in adult female ticks.

#### Effect of *RhEcR* gene silencing on the blood-feeding of adult female ticks

RNAi-mediated knockdown of *RhEcR* altered the development of adult female ticks. Gene silencing of *RhEcR* interfered with the morphological development (a consequence of the abnormal degeneration and remodeling in the integument during and after the blood meal) and resulted in unsuccessful blood-feeding (Fig. 5b). In contrast, gene silencing application of *RhUSP* had no effect on blood-feeding or morphological changes (Fig. 5e). Simultaneous silencing of *RhEcR* and *RhUSP* resulted in phenotypes similar to ds*RhEcR*-treated ticks (data not shown). The unsuccessful blood-feeding affected the normal growth and development of the female ticks. DsRhEcR-treated adult female ticks did not engorge successfully, had reduced weight compared to the control group, and females did not oviposit (Table 1). The data showed that RhEcR is essential for successful blood-feeding and tick development in *R. haemaphysaloides*.

**Table 1:** Statistical analysis of biological effects on dsRhEcR-treated and dsRhUSP-treated groups. It showed that the dsRhEcR-treated group had significant inhibition of feeding time, body weight, engorgement rate, oviposition rate, and egg-hatching rate compared to the dsLuciferase-treated group and that the dsRhUSP-treated group did not induce tick alterations

Group	Attachment rate in 24 h	Feeding period (days)	Engorged body weight (g)	Engorgement rate	Oviposition rate	Egg-hatching rate
dsEcR	50% (40/80)	7-10	0.0448 ± 0.0131*	0	0	0
dsLuciferase	47.5% (38/80)	7-8	0.2834 ± 0.0654	81.25% (65/80)	90% (18/20)	100%
dsUSP	62.5% (50/80)	7-8	0.2938 ± 0.0566**	85% (68/80)	95% (19/20)	100%

Student’s *t*-test was used to compare the weight of the dsRNA (target gene)-treated group and the control group

*P < 0.0001 (dsEcR vs. dsLuciferase)

**P < 0.0001 (dsUSP vs. dsLuciferase)

### Effect of *RhEcR* gene silencing on the degeneration of the tick salivary gland

Salivary gland degeneration was altered by RNAi-mediated knockdown of RhEcR. The treated ticks did not engorge, detach from the host, or oviposit. During the blood-feeding process, the salivary gland of dsRhEcR-treated ticks showed increased acini size compared to dsLuciferase-treated ticks. The cytoplasm in the granular acini formed coarse granules in dsRhEcR-treated ticks, but it was homogeneous in control ticks (Fig. 5g). In dsRhUSP-treated ticks, granular acini of the salivary gland showed normal morphology and a homogeneous cytoplasmic environment. At Fed 7, the acinar membrane shrank in both dsLuciferase- and dsUSP-treated group but not in the dsEcR-treated group. Therefore, the absence of detachment and histological data confirmed that dsRhEcR treatment had a crucial impact on degeneration of the salivary gland.

### Effect of *RhEcR* gene silencing on the apoptosis signaling pathway in ticks during salivary gland degeneration

**Fig. 6:**
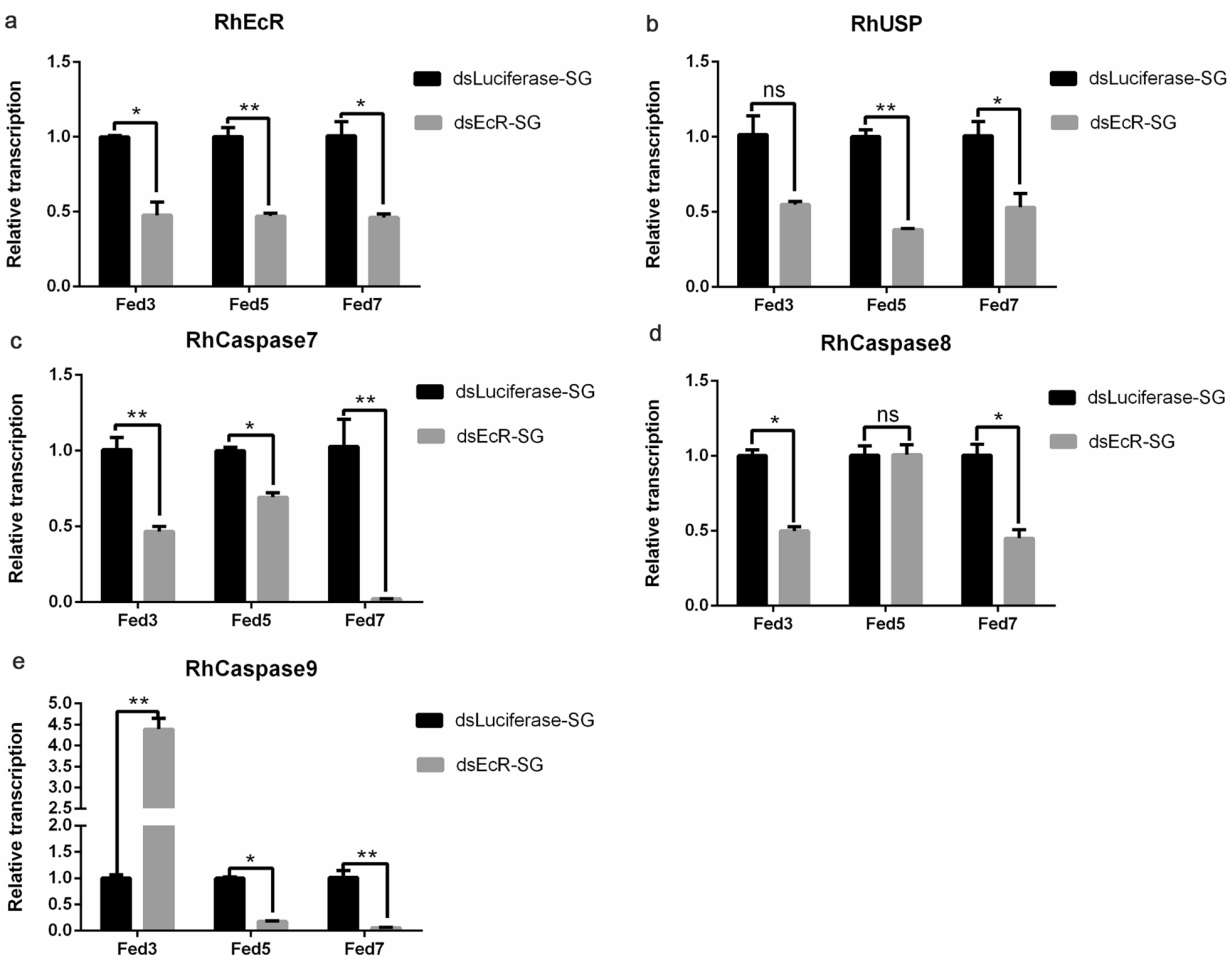
The transcriptional level of caspases was reduced in the dsRhEcR-treated group in *Rhipicephalus haemaphysaloides*. Using qRT-PCR, relative RNA levels of related genes were measured. The relative RNA levels of functional ecdysone receptors **a** *RhEcR* and **b** *RhUSP* and apoptosis signaling pathways **c** *RhCaspase7*, **d** *RhCaspase8*, and **e** *RhCaspase9* in the salivary gland were analyzed during the feeding period and in post-engorged female ticks. At the beginning of salivary gland degeneration, *RhCaspase7*, *RhCaspase8*, and *RhCaspase9* were downregulated. At all of the test time points, the *RhCaspase7* RNA level was lower than that in the control group. Treatment with dsRhEcR downregulated the RNA levels of apoptosis-related genes in the salivary gland. This showed inhibition and degeneration of the salivary gland in dsRhEcR-treated ticks. The data represent the mean ± SD of the experiments (10 ticks/time point) performed in triplicate and normalized to EF1α, and two-tailed Student’s *t*-tests were used to identify significant differences between groups (RhEcR-Fed3: t = 5.815, df = 2, P = 0.0283; RhEcR-Fed5: t = 11.68, df = 2, P = 0.0072; RhEcR-Fed7: t = 6.264, df = 2, P = 0.0246; RhUSP-Fed3: t = 4.247, df = 2, P = 0.0512; RhUSP-Fed5: t = 12.19, df = 2, P = 0.0067; RhUSP-Fed7: t = 9.101, df = 2, P = 0.0119; RhCaspase7-Fed3: t = 4.916, df = 2, P = 0.0390; RhCaspase7-Fed5: t = 8.542, df = 2, P = 0.0134; RhCaspase7-Fed7: t = 5.716, df = 2, P = 0.0293; RhCaspase8-Fed3: t = 7.832, df = 2, P = 0.0159; RhCaspase8-Fed5: t = 0.2845, df = 2, P = 0.8028; RhCaspase8-Fed7: t = 6.299, df = 2, P = 0.0243; RhCaspase9-Fed3: t = 11.69, df = 2, P = 0.0072; RhCaspase9-Fed5: t = 26.66, df = 2, P = 0.0014; RhCaspase9-Fed7: t = 6.933, df = 2, P = 0.0202, ns *P* > 0.05, *P < 0.05, **P < 0.01)

**Fig. 7:**
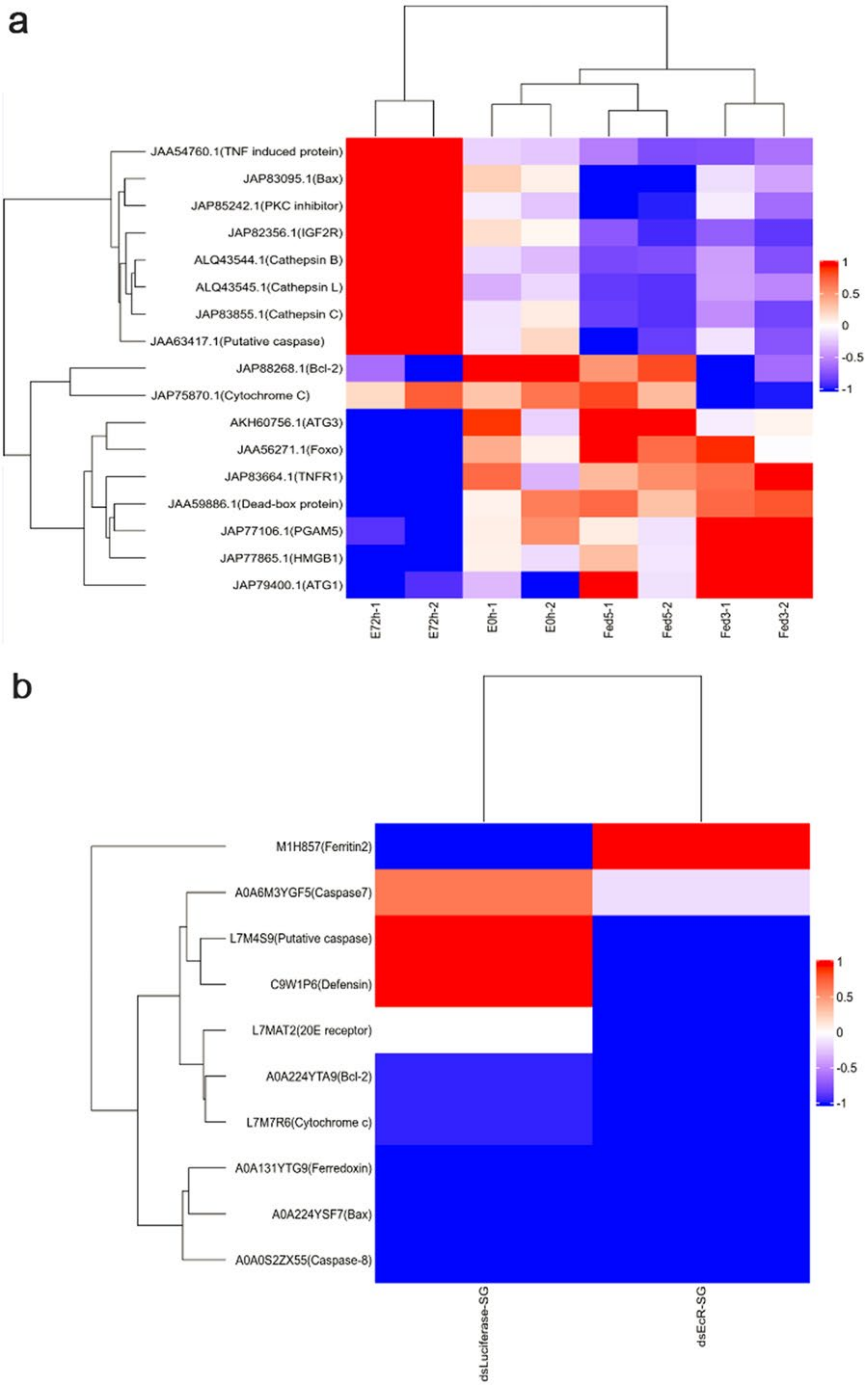
The translational level of caspases was reduced in the dsRhEcR-treated group in *Rhipicephalus haemaphysaloides*. The quantitative analysis of salivary gland proteome changes was performed by TMT isobaric labeling followed by shotgun analysis. **a** A heat map was plotted based on the set of apoptosis-related, autophagy-related, and other proteins that were significantly decreased and increased (*P* < 0.05) at least 1.2-fold from the E 72h group compared with the E 0h group. **b** The heat map was plotted based on the set of apoptosis-related proteins that were significantly decreased (*P* < 0.05) at least 1.2-fold and increased (*P* < 0.05) at least tenfold from the dsRhEcR-treated group compared with the dsLuciferase-treated group

Because the apoptosis signaling pathway plays a key role in the degeneration of the salivary gland, the effects of RhEcR on this pathway were analyzed based on the RNA levels of *RhEcR*, *RhUSP*, *RhCaspase7*, *RhCaspase8*, and *RhCaspase9* (Fig. 6a–e). The RNA levels of the *RhEcR*, *RhUSP*, and *RhCaspase7* genes were significantly reduced in dsRhEcR-treated females compared to the dsLuciferase-treated females (Fig. 6a–c). However, *RhCaspase8* was significantly reduced at Fed 3 and Fed 7 but not at Fed 5, and *RhCaspase9* was higher at Fed 3 and lower at Fed 5 and Fed 7 in the dsRhEcR-treated ticks than in the control group (Fig. 6d and e). The RNA levels of *RhCaspase7*, *RhCaspase8*, and *RhCaspase9* were lower than those of the control group at Fed 7, when salivary gland degeneration was underway. Additionally, in the salivary gland of normal females, the RNA levels of the *RhEcR*, *RhUSP*, *RhCaspase7*, *RhCaspase8*, and *RhCaspase9* genes were increased in the fast blood-feeding and post-engorgement periods. Comparative analysis of salivary gland proteomes between the dsRhEcR-treated group and dsLuciferase-treated group showed that apoptosis-related proteins were altered after gene silencing. The heat map in Fig. 7b shows that caspases and proteins related to the intrinsic pathway of apoptosis decreased in the *dsRhEcR*-treated group compared to the control group. In addition, proteomic analyses during salivary gland degeneration showed that the expression of the apoptosis-related protein caspases increased gradually. The heat map in Fig. 7a shows an alteration in expression of apoptosis- and autophagy-related proteins in normal female ticks during and after the feeding process. These data indicate the participation of RhEcR in salivary gland degeneration by regulation of caspases in the apoptosis signaling pathway.

### Heterologous co-expression of RhEcR and RhUSP induced cell death by apoptotic mechanisms

**Fig. 8:**
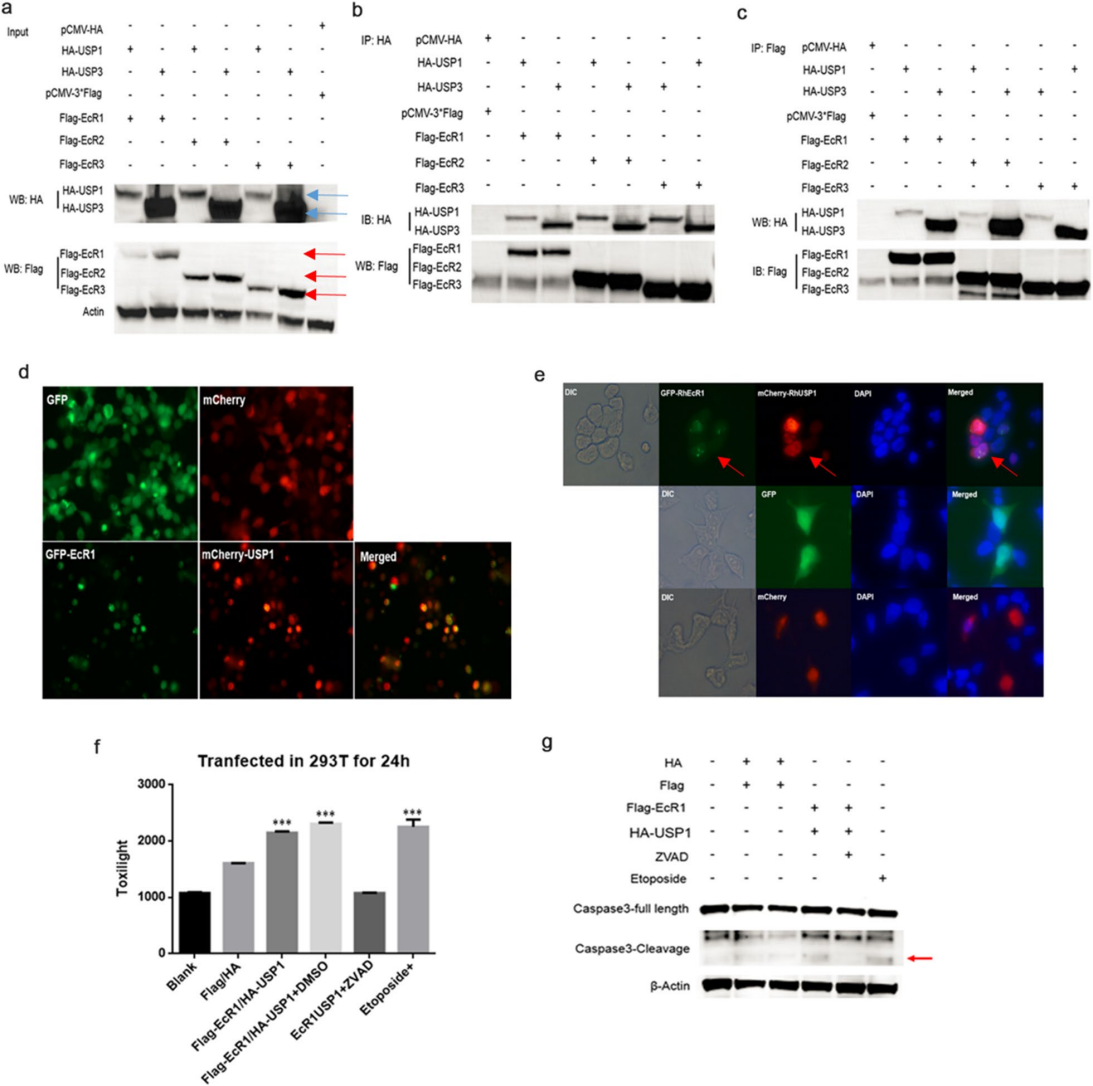
Co-expression of RhEcR and RhUSP induced cell death of HEK293T cells by apoptotic signals. **a**, **b**, **c** The results showed that different isoforms of RhEcR can interact with different RhUSP isoforms in HEK293T. **a** The expression of each isoform of Flag-RhEcR and HA-RhUSP in HEK293T cells. **b** Isoforms of Flag-RhEcR were detected by co-immunoprecipitation of HA-RhUSP. **c** Isoforms of HA-RhUSP were detected by co-immunoprecipitation of Flag-RhEcR. **d** The complex of GFP-RhEcR1 and mCherry-RhUSP1 induced cell death in HEK293T cells. GFP, mCherry, and GFP-RhEcR1 together with mCherry-RhUSP1 were transfected in HEK293T for 24 h. Images were obtained using a fluorescence microscope. **e** GFP-RhEcR1 and mCherry-RhUSP1 were co-located in the nucleus. GFP-RhEcR1 and mCherry-RhUSP1 were co-expressed in HEK293T for 24 h. Images were obtained using a fluorescence microscope. **f**, **g** The complex of Flag-RhEcR1 and HA-RhUSP1 induced cell death through the activation of the apoptosis pathway in HEK293T. Cleaved caspase-3 was detected during co-expression of Flag-RhEcR1 and HA-RhUSP1 and etoposide was induced. The agonist of apoptosis, etoposide, and co-expression of Flag-RhEcR1 and HA-RhUSP induced cell death. Co-expression of Flag-RhEcR1 and HA-RhUSP1 together with the caspase inhibitor, zVAD, inhibited cell death. In **f**, the data represent the mean ± SD of the experiments performed in triplicate one-way ANOVA multiple Comparisons were used to identify significant differences between groups (***P < 0.001) (ANOVA: Flag-EcR1/HA-USP1 vsFlag/HA, *F* (2, 6), *P* < 0.001; Flag-EcR1/HA-USP1 vs Flag-EcR1/HA-USP1/DMSO, *F* (2, 6), *P* > 0.9999; Flag-EcR1/HA-USP1/DMSO vs Flag-EcR1/HA-USP1/ZVAD, *F* (2, 6), *P* < 0.001; Blank vsEtoposide+, *F* (2, 6), *P* < 0.001)

In order to further explore the interaction between RhEcR and RhUSP, HEK293T cells were transfected with plasmids for each isoform of RhEcR and RhUSP. The co-expression of one isoform of each protein of the complex permitted testing of the binary interaction between the RhEcR and RhUSP isoforms. The combination of the co-expression of RhEcR-tagged Flag and RhUSP-tagged HA was analyzed. As shown in Fig. 8a, each isoform of Flag-RhEcR (red arrows) and each isoform of HA-RhUSP (blue arrows) were co-expressed in HEK293T cells. To identify the interaction between RhEcR and RhUSP, a co-immunoprecipitation assay was performed using anti-HA or anti-Flag immunomagnetic beads. The RhEcR-RhUSP complexes were detected in all conditions tested, as demonstrated by immunoblot analyses with antibodies directed against HA-Tag (Fig. 8b) or Flag-tag (Fig. 8c).

The role of the RhEcR1-RhUSP1 complex in cell death was also analyzed by transfection of HEK293T with GFP-RhEcR1 and mCherry-RhUSP1 constructs. At 24 h post-transfection, it was observed that overexpression of RhEcR1-RhUSP1 resulted in apoptosis (Fig. 8d and f). Also, the intracellular localization showed that the GFP-RhEcR1 and mCherry-RhUSP1 complex was located exclusively in the nucleus of the cells (Fig. 8e; indicated by arrows). The role of apoptosis in eliciting this cell death was demonstrated by the detection of cleaved caspase-3 in cells transfected with the complex of Flag-RhEcR1 and HA-RhUSP1 (Fig. 8g). To confirm the specificity of caspase-3 cleavage and activation of apoptotic cell death, the RhEcR1-RhUSP1 effect in cell death was inhibited with zVAD, a caspase inhibitor (Fig. 8f and g).

## Discussion

In *R. haemaphysaloides* females, the ecdysteroid functions in the regulation of the salivary gland, production of oogenesis, and oviposition [16]. In this study, an ecdysteroid receptor, a heterodimer of EcR and USP, was identified in the salivary gland of *R. haemaphysaloides* (Fig. 1a, b). The differences among the three isoforms of RhEcR or between two isoforms of RhUSP was mostly in the variable N-terminal A/B domain. In vertebrates, the subtypes of a particular nuclear receptor (NR) are products of different genes (e.g., thyroid hormone receptors TRα and TRβ) [40]. In arthropods, receptor isoforms are products of alternative splicing and/or alternative promoters of a single gene [41]. In *R. haemaphysaloides*, it is less clear whether isoforms of RhEcR or isoforms of RhUSP are products of a single gene or different genes due to a lack of genomic information. Nevertheless, homologous genes were found to be present in only one copy in the genome of six ixodid tick species (Additional file 1: Figure S4) using an analysis method [42], suggesting the presence of only one copy in *R. haemaphysaloides*.

During blood-feeding and post-engorgement, RhEcR and RhUSP showed tissue specificity and a wide temporal distribution profile (Fig. 2a and b). RhEcR/ RhUSP expression was higher in the Ov, SG, and Gut. In the post-engorgement stage, under physiological conditions, the Ov undergoes a maturation process for egg production and oviposition, while SG undergoes a degenerative process. Therefore, in *R. haemaphysaloides,* the ecdysone receptor has an important role for the ovary and salivary gland, similar to that in insects [43] and other ticks species [44]. Meanwhile, the highest amount of 20E in SG was observed at the beginning of the degeneration. The 20E gradually decreased during the degenerative process (Fig. 4a), and this was correlated with the expression of *RhEcR*. In a similar manner*,* the 20E concentration also increased during the rapid phase of engorgement, but it was stable during the feeding period and decreased after detachment and salivary gland degeneration in *A. hebraeum* [21]. In the hemolymph, the 20E concentration increased during the rapid feeding phase and maintained the concentration until the last analysis point (Fig. 4b). The 20E is a small, lipid-soluble molecule that can access cells easily, but the different concentrations observed in the different tissues suggest that 20E absorption may be tissue-selective. Further studies are needed to analyze the relationship between the 20E concentration and the function of EcR/USP in order to improve our understanding of the salivary gland regulatory physiology. In addition, these data may support the development of new tick and tick-borne pathogen control methods. In the same way, the temporal transcription profile of each isoform was determined in the female salivary gland. During the blood-sucking process and until 24 h post-engorgement, the *RhEcR2* RNA level was higher than that of *RhEcR1;* however, subsequently, the *RhEcR1* RNA level was higher than that of *RhEcR2* (Fig. 3a). These profiles suggest that *RhEcR2* plays an important role in the maturation of the salivary gland and that *RhEcR1* influences the degeneration process of the salivary gland. This EcR profile is similar to that observed in *A. hebraeum* [45]. However, the protein levels of RhEcR isoforms decreased gradually after 24 h post-engorgement (Fig. 3b). The transcription levels and translation levels are not necessarily the same, but the trend is usually similar. In addition, the two isoforms of *RhUSP* were detected during all time points, but *RhUSP1* had a higher RNA level than *RhUSP3* (Fig. 3c). These data suggest that RhEcR/*RhUSP* play a role in the process of salivary gland degeneration.

RNAi is the most effective method for identifying gene functions in ticks [46]. Therefore, RNAi was used to study the physiological roles of RhEcR and RhUSP. *RhEcR* silencing produced a significant phenotype alteration in ticks during blood-feeding compared to the *RhUSP* silencing and the control groups. The dsRhEcR-treated ticks did not engorge and detach and failed to maintain development. As a consequence, the tick weights were significantly reduced, and females did not oviposit (Fig. 5b and Table 1), corroborating the role of EcR as previously observed in insects [47, 48] and other ticks [49, 50]. While these data are promising, further work is still needed to establish the precise role of each RhEcR isoform in tick physiology. Apoptosis is an essential mechanism for organ development and is documented in several species. In ticks, the homoeostasis of the SG, Gut, Ov, and other organs is maintained through apoptosis [14, 17, 51, 52]. Generally, when ticks detach from the host, the salivary glands began to gradually degenerate. In the dsEcR-treated group, ticks did not detach from the host, so the degeneration of the salivary gland must have been inhibited, and no shrinkage of the acinar membrane was observed. EcR/USP is a major signal for the alteration in gene expression patterns needed to control cell death during tissue remodeling activity in ticks and insects. However, the exact mechanism is unclear [21, 53, 54]. To determine the effect of RhEcR on apoptosis during the process of salivary gland degeneration, the expression of apoptosis-related genes was analyzed. In dsRhEcR-treated female ticks, the RNA levels of *RhCaspase9* and *RhCaspase7* were downregulated during the early stage of salivary gland degeneration (Fig. 6e, c). These data were supported by a salivary gland proteome analysis. As shown by the heat map in Fig. 7a, the expression of a set of apoptosis-related proteins was significantly lower in E 0 h ticks than E 72h ticks, indicating that the expression of apoptosis-related proteins increases during salivary gland degeneration. In addition, the heat map of RNAi-treated ticks showed a lower abundance of apoptosis-related proteins in the dsRhEcR-treated group than in the control group (Fig. 7b). This indicated that dsRhEcR reduced the expression of apoptosis proteins, particularly caspases and proteins in the intrinsic apoptotic pathway. In this study, it was not possible to determine the cleavage of caspases due to the lack of specific and effective antibodies against ticks. Although cleavage of caspases was not detected, a reduction in the total protein level of caspases suggests that apoptosis is involved in salivary gland degeneration. The data support the role of RhEcR and RhUSP signaling in the process of salivary gland degeneration.

To analyze the interaction between the RhEcR and RhUSP, we performed a co-transfection of HEK293T cells with plasmids containing different isoforms of *RhEcR* and *RhUSP*. The HEK293T cell line has previously been used to analyze different molecular mechanisms of apoptosis induction [52], including transfection with EcR/USP expression plasmids [55, 56]. This in vitro system made it possible to observe that each RhEcR isoform could interact as a heterodimer with any of the RhUSP isoforms. The data also showed that heterologous co-expression of RhEcR1/RhUSP1 induced a process of apoptotic cell death in the HEK293T cells. These results demonstrate the action of the ecdysone receptor, RhEcR/RhUSP, in apoptosis during *R. haemaphysaloides* salivary gland degeneration. It is generally well known that the activity of the EcR/USP complex is 20E-dependent. The ecdysteroids, invertebrate steroid hormones, elicit genomic but also non-genomic effects [27]. Classically, ligand binding was found to induce the ability of nuclear receptors to modulate the transcription rate of target genes (genomic effects), which led to considering them as ligand-activated transcription factors, and non-genomic effects are independent of gene transcription or protein synthesis and involve steroid-induced modulation of cytoplasmic or cell membrane-bound regulatory proteins [57]. However, the RhEcR1/RhUSP1 complex triggered apoptosis without 20E in HEK293T. Therefore, how is RhEcR1/RhUSP1-mediated apoptosis induced without 20E? Perhaps the complex functioned like non-genomic effects by modulating related proteins in the apoptosis pathway. Further research is needed in this area. The mechanisms of apoptosis are important, as it is a critical component of salivary gland degeneration and tick physiology. The ability to disrupt or inhibit the development of ticks using *EcR* silencing or EcR inhibitors can advance our knowledge of tick physiology. Strategies that interfere with the apoptotic signaling pathways may aid in the development of new tick control methods and can be useful in reducing diseases caused by ticks in animals and humans. In conclusion, *EcR* is a critical gene controlling the normal development of ticks, and it is a promising target for controlling ticks and tick-borne diseases.

## Conclusions

The ecdysteroid receptor, a heterodimer of RhEcR and RhUSP, was identified in *R. haemaphysaloides*, and the role of the ecdysteroid receptor in the apoptosis process was characterized. Our findings enhance our understanding of the processes involved in salivary gland development and degeneration. RhEcR plays a key role in growth and development during blood-feeding and in the regulation of salivary gland degeneration in females. The participation of the RhEcR/RhUSP in the apoptosis process was further corroborated by the induction of apoptotic cell death in HEK293T expressing the RhEcR1 and RhUSP1 complex.

## Supplementary Information

**Additional file 1: Figure S1.** Alignment of the deduced amino acid sequences of *Rhipicephalus haemaphysaloides*-EcR; *Amblyomma americanum*-EcR; *Ixodes scapularis*-EcR; *Ornithodoros moubata*-EcR; *Bombyx mori*-EcR; *Drosophila melanogaster*-EcR; *Danio rerio*-LXRα; *Homo sapiens*-LXRα; *Mus musculus*-LXRβ. All species had a DBD domain and an LBD domain, and the DBD domain had two conserved C4-type zinc finger motifs. Especially, the position of four cysteine residues in the ZF_C4 was highly conservative. In arthropods, the DBD and LBD domains were relatively conserved. **Figure S2.** Alignment of the deduced amino acid sequences of *Rhipicephalus haemaphysaloides* USP; *Amblyomma americanum*-USP; *Ixodes scapularis*-USP; *Ornithodoros moubata*-USP; *Bombyx mori*-USP; *Drosophila melanogaster*-USP; *Danio rerio*-USP; *Homo sapiens*-RXR; *Mus musculus*-RXR. Invertebrates and vertebrates both had a DBD domain and an LBD domain as EcR. Moreover, the DBD domain of USP was highly conserved not only in invertebrates but also in vertebrates, which was different from EcR. **Figure S3.** Phylogenetic tree constructed using amino acid sequences of the ecdysteroid receptor heterodimeric complex, EcR (a) and USP (b) of *Rhipicephalus haemaphysaloides.* **Figure S4.** Results of a BLAST search of *RhEcR* and *RhUSP* sequences against genomic sequences of six tick species. The query was *RhEcR* and *RhUSP* gene nucleotide sequences in a search against genomic sequences of six tick species (*Ixodes persulcatus*, *Haemaphysalis longicornis*, *Dermacentor silvarum*, *Hyalomma asiaticum*, *Rhipicephalus sanguineus*, *Rhipicephalus microplus* and *Ixodes scapularisis*). **Figure S5.** Temporal and spatial distribution of *RhEcR *and *RhUSP* in *Rhipicephalus haemaphysaloides*. Tissues were dissected from female ticks. Using qRT-PCR, relative RNA levels of *RhEcR* and *RhUSP* were measured at each time point. **a** In the period of blood-feeding, the expression of *RhEcR2* was higher than *RhEcR1* before E 24 h. After E 24 h, the expression of *RhEcR1 *was higher than *RhEcR2*. **b** Two isoforms of *RhUSP* were detected during all time points, but the level of *RhUSP1* was high and *RhUSP3* was very low. The data represent the mean ± SD of the experiments (10 ticks/time point) performed in triplicate and normalized to EF1α. **c** The translation of RhEcR isoforms was detected with anti-mouse RhEcR polyclonal antibody. After E 24 h, the expression of all isoforms of RhEcR gradually decreased. **Table S1** Oligonucleotide sequences and names of the primers used for qRT-PCR, RNAi and cloning of *Rhipicephalus haemaphysaloides* genes. **Table S2** Accession numbers for sequences of EcR/LXR and USP/RXR in vertebrates and invertebrates.

**Additional file 2: Dataset S1.** Contigs of the *Rhipicephalus haemaphysaloides* salivary gland for *RhEcR* cloning. **Dataset S2.** Contig of the *Rhipicephalus haemaphysaloides* salivary gland for *RhUSP* cloning.

## Data Availability

All data generated or analyzed during this study are included in this published article and its additional files. The datasets supporting the findings of this article are included within the paper. The proteomics data have been deposited in the iProX database (https://www.iprox.cn/page/HMV006.html) with accession number IPX0003561000.

## Abbreviations

*R. haemaphysaloides*: *Rhipicephalus haemaphysaloides*
RhEcR: *Rhipicephalus haemaphysaloides* ecdysone receptor
RhUSP: *Rhipicephalus haemaphysaloides* ultraspiracle
RXR: Retinoid X receptor
DBD: DNA-binding domain
LBD: Ligand-binding domain
20E: 20-Hydroxyecdysone
ORF: Open reading frame
PI: Isoelectric point
Mw: Molecular weight
qRT-PCR: Quantitative real-time polymerase chain reaction
SG: Salivary gland
FB: Fat body
Ov: Ovary
Sy: Synganglion
RNAi: RNA interference
EIA: Enzyme immunoassay
KLH: Keyhole limpet hemocyanin
PcAb: Polyclonal antibodies
SDS-PAGE: Sodium dodecyl sulfate–polyacrylamide gel electrophoresis
NLS: Nuclear localization signal
NES: Nuclear export signal
HE: Hematoxylin–eosin
PMSF: Phenylmethanesulfonyl fluoride
HRP: Horseradish peroxidase
